# Chemistry and Biological Activity of Alkaloids from the Genus *Lycoris* (Amaryllidaceae)

**DOI:** 10.3390/molecules25204797

**Published:** 2020-10-19

**Authors:** Lucie Cahlíková, Kateřina Breiterová, Lubomír Opletal

**Affiliations:** ADINACO Research Group, Department of Pharmaceutical Botany, Faculty of Pharmacy, Charles University, Heyrovského 1203, 500 05 Hradec Králové, Czech Republic; breiterk@faf.cuni.cz (K.B.); opletal@faf.cuni.cz (L.O.)

**Keywords:** Amaryllidaceae, *Lycoris*, *Lycoris radiata*, folk medicine, alkaloids, biological activity

## Abstract

*Lycoris* Herbert, family Amaryllidaceae, is a small genus of about 20 species that are native to the warm temperate woodlands of eastern Asia, as in China, Korea, Japan, Taiwan, and the Himalayas. For many years, species of *Lycoris* have been subjected to extensive phytochemical and pharmacological investigations, resulting in either the isolation or identification of more than 110 Amaryllidaceae alkaloids belonging to different structural types. Amaryllidaceae alkaloids are frequently studied for their interesting biological properties, including antiviral, antibacterial, antitumor, antifungal, antimalarial, analgesic, cytotoxic, and cholinesterase inhibition activities. The present review aims to summarize comprehensively the research that has been reported on the phytochemistry and pharmacology of the genus *Lycoris*.

## 1. Introduction

Plants of the Amaryllidaceae family, which consists of about 85 genera and 1100 species, are distributed over the tropical and warm regions of the world. They have been extensively used as folk medicines to treat various diseases in many countries and areas [1,2,3]. Chemically, the Amaryllidaceae family is known for its unique alkaloid constituents, named Amaryllidaceae alkaloids (AAs), which display a wide range of biological activities including acetylcholinesterase (AChE) and butyrylcholinesterase (BuChE) inhibition effects, and antitumor, antifungal, antibacterial, antiviral, and antimalarial properties [1,4,5,6]. The most known representative of AAS is galanthamine, which is currently used for the treatment of early and intermediate states of Alzheimer’s disease (AD) [7].

The present review summarizes phytochemical studies carried out on the genus *Lycoris*, focusing on the occurrence, isolation, identification and biological activities of its alkaloids. *Lycoris* species have been used for a long time in traditional medicine.

## 2. Genus Lycoris: Occurrence, Ethnobotany

The genus *Lycoris* Herbert consists of about 20 species which are distributed in the moist warm temperate woodlands of eastern Asia, as in China, Korea, Japan, Taiwan, and the Himalaya [8,9]. The genus was established by Herbert in 1821 [10], and *L. aurea* has been assigned as the type species. In the mid-19th to early 20th centuries, nine new species were published by various European authors (*L. africana*, *L. straminea*, *L. sewerzowii*, *L. squamigera*, *L. sanguinea*, *L. terraccianii*, *L. sprengeri*, *L. incarnata* and *L. argentea*). In the first half of the 20th century three new species from Japan were described (*L. albiflora*, *L. koreana* and *L. kiusiana*) [10]. After that, American botanist Hamilton P. Traub recorded 10 new taxa (two with Moldenke) of *Lycoris*, based mainly on materials introduced from China, Japan and cultivated in American gardens (*L. rosea*, *L. haywardii*, *L. caldwelii*, *L. houdyshelii* and *L.* × *woodii*, *L. chinensis*, *L. elsiae*, *L.* × *lajolla*, *L.* × *jacksoniana*, *L. josephinae*) [10]. American horticulturist Hayward published, in 1957, details of a new species, *L. traubii*. About 15 years later, Chinese botanists recorded four new species of *Lycoris* from China: *L. longituba*, *L. anhuiensis*, *L. guangxiensis*, and *L. shaanxiensis* [10]. In the 1990s, new species from Korea were described by Korean botanists (*L. flavescens*, and *L. chejuensis*) [8,10]. However, some of these species have been reduced to synonyms, some to intraspecific rank, and still others are cultivars or hybrids, since *Lycoris* species easily hybridize with each other [10]. Plants of the genus *Lycoris* are extensively cultivated as ornamental plants, especially in China and Japan, and also in other warm temperate regions of the world. Over 230 cultivars have been selected for garden use. Currently, The Plant List accepts twenty-three *Lycoris* species [11].

Plants of the genus *Lycoris* have been used for a long time in traditional medicine. The bulbs of *L. radiata* were used as a traditional Chinese herbal medicine to treat sore throats, carbuncle, cancer, suppurative wounds, poliomyelitis, mastitis, tympanitis, ulcers, and neurodegenerative diseases like AD, but generally, *L. radiata* has been considered to be toxic [12,13,14,15,16]. In the Compendium of Materia Medica in China, it is recorded that *L. radiata* can also be used as an antidote to poison, relieve inflammation, alleviate pain, and act as a diuretic drug [17]. *L. sprengeri* is mainly distributed in the lower reaches of the Yangtze River, and its bulbs have also been used in Chinese medicine for the treatment of similar diseases as *L. radiata* [18]. The crushed bulbs of *L. aurea*, which is endemic to the southwest district of Hubei Province in China, have been used as a poultice for treating burns, scalds and ulcers [19].

## 3. Phytochemistry of the Genus Lycoris

**Figure 1:**
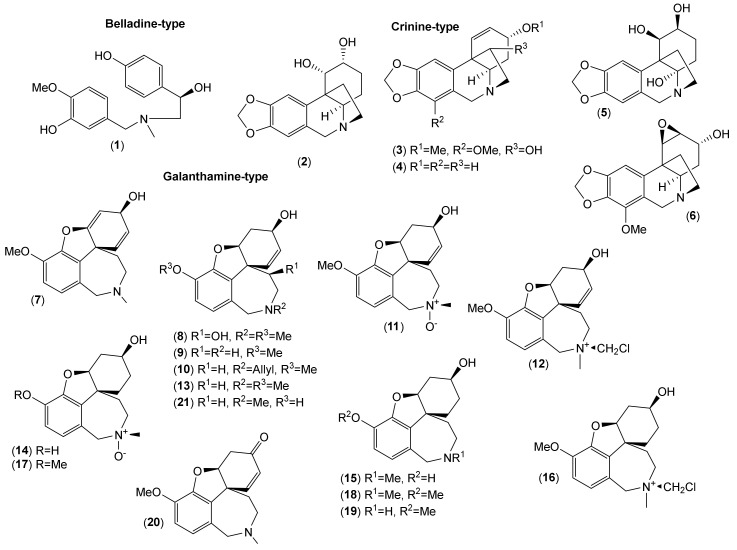
Amaryllidaceae alkaloids of belladine-, crinine-, and galanthamine-type reported in *Lycoris* species.

**Figure 2:**
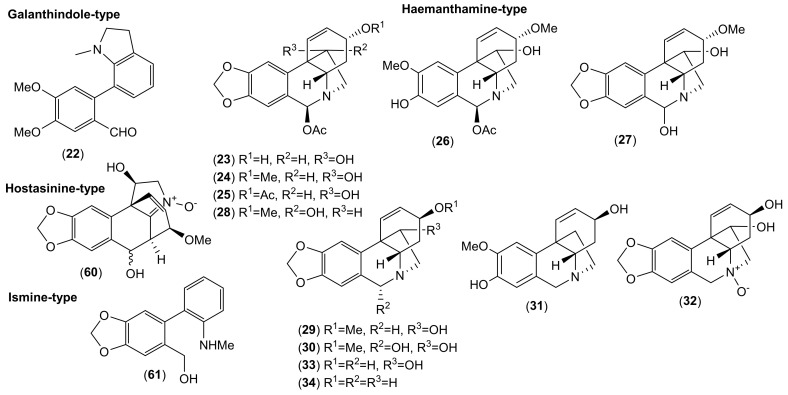
Amaryllidaceae alkaloids of galanthindole-, haemanthamine-, hostasinine-, and ismine-type reported in *Lycoris* species.

**Figure 3:**
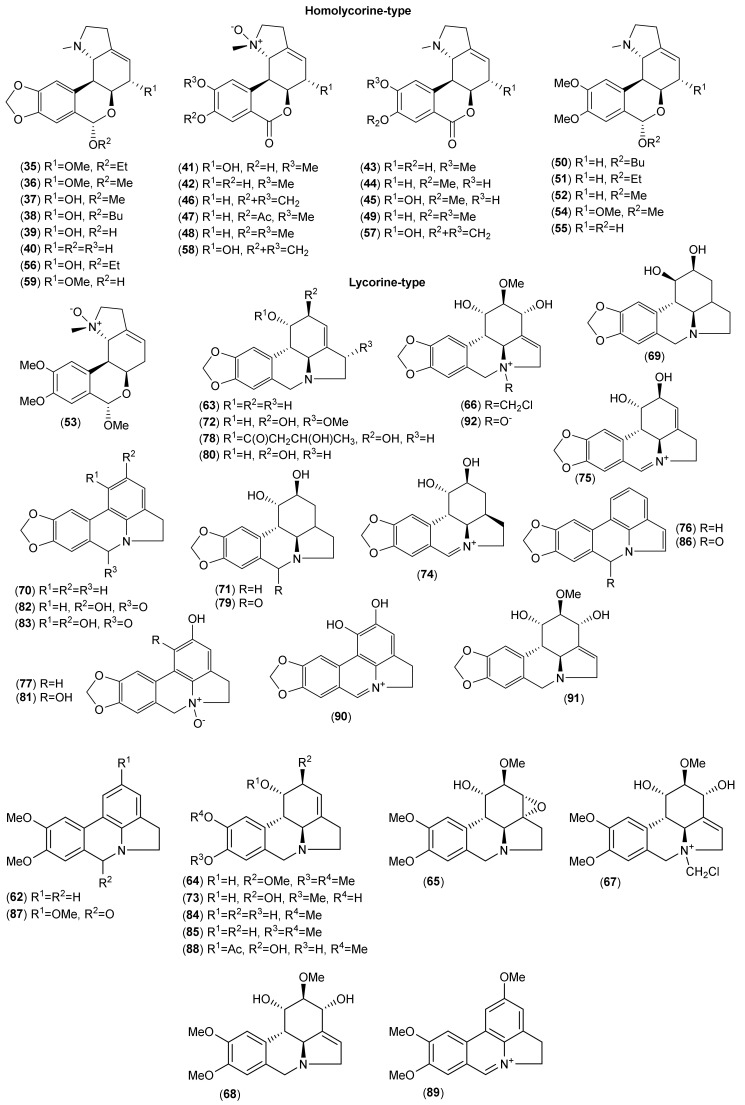
Amaryllidaceae alkaloids of homolycorine- and lycorine-type reported in *Lycoris* species.

**Figure 4:**
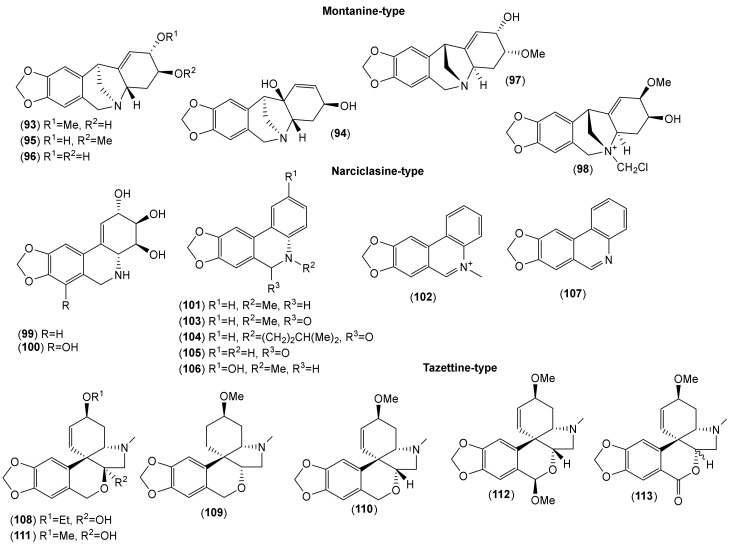
Amaryllidaceae alkaloids of montanine-, narciclasine-, and tazettine-type reported in *Lycoris* species.

**Figure 5:**
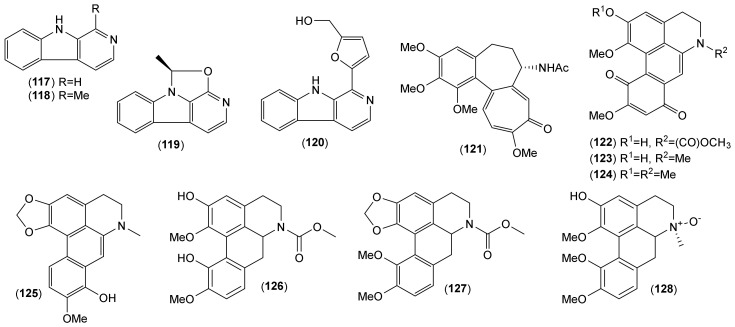
Alkaloids of other structural types isolated from *Lycoris* plants.

Of the accepted 23 *Lycoris* species, fourteen have been chemically investigated (Table 1). Major attention within the reported phytochemical studies has been given to the study of alkaloids, since they are the most studied constituents of this genus, and only little attention has been given to other components. The AAs are largely restricted to the family Amaryllidaceae, specifically the subfamily Amaryllidoideae [20]. They are derived from the aromatic acids phenyalanine and tyrosine, which are used to produce key intermediates in the biosynthesis of the AA 4′-*O*-methylnorbelladine [21,22]. According to the name of this key intermediate, this biosynthetic pathway of AAs is called the norbelladine pathway [23]. Recently, several review articles provide detailed coverage of the biosynthesis of AAs [1,23,24,25,26], and thus we will not repeat this in the current review. Altogether, 116 AAs of various structural types have been isolated in either pure form or identified by different analytical methods (e.g., GC-MS or HPLC-MS) in the studied *Lycoris* plants (Table 1; Figure 1, Figure 2, Figure 3, Figure 4 and Figure 5). The reported alkaloids belong to the belladine (**1**), crinine (**2**–**6**), galanthamine (**7**–**21**), galanthindole (**22**), haemanthamine (**23**–**34**), homolycorine (**35**–**59**), hostasinine (**60**), ismine (**61**), lycorine (**62**–**92**), montanine (**93**–**98**), narciclasine (**99**–**107**) and tazettine (**108**–**116**) structural types (Table 1, Figure 1, Figure 2, Figure 3, Figure 4 and Figure 5). Moreover, 12 alkaloids (**117**–**128**) of other structural cores have been reported in some *Lycoris* species (Table 1).

The most studied *Lycoris* species, *L. radiata*, also called spider lily, is a horticultural plant widely distributed in the south of China, Vietnam, Korea, Nepal and Malaysia [14,15,27]. Altogether 79 Aas have been either identified or isolated from either the bulbs or flowers of this plant (Table 1). This species is rich in galanthamine-, haemanthidine-, homolycorine- and lycorine-type Aas. From the bulbs of *L. radiata*, the alkaloid colchicine (**128**) has recently been isolated; this is also derived from tyrosine, as are AAs, but it is metabolite typical of the genus *Colchicum* [28]. The question is whether it is a real product of the *Lycoris* plant, and not a product of some contamination with another plant, since this is the only report of this alkaloid-type within the whole Amaryllidaceae family. The same is also true for the reports of further alkaloids of other structural types (**117**–**120** and **122**–**128**; Table 1). The natural existence of an *N*-chloromethyl moiety in galanthamine-, lycorine-, and montanine-type AAs, such as in *N*-chloromethylgalanthamine (**12**), *N*-chloromethyllycoramine (**16**), *N*-chloromethylungiminorine (**66**), *N*-chloromethylnarcissidin (**67**), and lycolongirine C (**98**) isolated from bulbs of *L. longituba, L. radiata,* and *L. sprengeri* must be reinvestigated, because when halogenated solvents are used during the isolation process, this can result in the formation of *N*-chloromethyl alkaloid derivative artifacts [29]. In the course of phytochemical study of the mentioned *Lycoris* species, dichloromethane has been used for extraction and column chromatography of the alkaloids, and this can be the explanation for the isolation of the mentioned compounds [30]. Either HPLC/MS or GC/MS analysis should be used for analysis of the alkaloidal extract before the separation process. Then, the presence of isotopes of ^35^Cl and ^37^Cl in the mass spectra of the separated compounds can indicate the natural presence of *N*-chlormethylated derivatives. To avoid the possible formation of the mentioned artifacts, ethylacetate is recommended to be used for the preparation of the alkaloidal extract instead of halogenated solvents such as dichloromethan or chloroform. Another question is the natural presence of the butoxy-moiety at the C6 position in homolycorine-type compounds such as 2*α*-hydroxy-6-*O*-*n*-butyloduline (**38**) and *O*-*n*-butyllycorenine (**50**), both isolated from *L. aurea* in a study from 2014, due to the reactivity of the carbonyl and hemiacetal groups [29]. The authors stated the use of 95% alcohol for the total extraction of fresh bulbs, but the exact type of alcohol was not specified at the beginning of the isolation process [31]. It can be assumed that butanol was used for this initial isolation step. To avoid discussion and speculation as to whether the isolated product is really a natural compound and not an isolation artefact, it is necessary to specify the solvents used within all steps of the phytochemical study.

Some authors also discuss the natural origin of the methoxy- and ethoxy-moieties in homolycorine-type AAs [29], when either methanol or ethanol are used during separation. It should also be noted that some AAs reported in the genus *Lycoris*, such as 1,2,11,12-tetradehydrogalanthamine (**7**), 3-hydroxylatifaliumin C (**114**), dihydrolatifaliumin C (**115**), and latifaliumin C (**116**) [32,33] have been only tentatively identified on the basis of their EI mass spectra. Further isolation and spectroscopic studies of these compounds are necessary for their unambiguous identification and structure determination. A complete overview of AAs identified and isolated from *Lycoris* species is summarized in the following table (Table 1).

**Table 1:** Alkaloids reported in the genus *Lycoris*.

	*L. albiflora*	*L. aurea*	*L. caldwelii*	*L. chinensis*	*L. guangxiensis*	*L. haywardii*	*L. incarnata*	*L. longituba*	*L. radiata*	*L. radiata* var. *pumila*	*L. sanguinea*	*L. sprengeri*	*L. squamigera*	*L. traubii*
*Belladine-type*														
2*R*-Hydroxy-*N*,*O*-dimethylnorbelladine (**1**)													[34]	
*Crinine-type*														
Amabiline (**2**)									[28]					
Ambelline (**3**)		[32]							[32]					
Crinine (**4**)					[35]									
Crinamabine (**5**)					[32]				[32]					
Crinamidine (**6**)		[32]			[32]				[32]					
***Galanthamine-type***														
1,2,11,12-Tetradehydrogalanthamine * (**7**)									[32]					
11β-Hydroxygalanthamine (**8**)								[30]	[28]					
*N*-Norgalanthamine/*N*-Demethylgalanthamine (**9**)		[31,32]			[32,35]			[30]	[32]				[34]	
*N*-Allylnorgalanthamine (**10**)					[35]				[28]					
Galanthamine *N*-oxide (**11**)							[36]		[37,38]					
*N*-(Chloromethyl)galanthamine (**12**)		[31]						[30]						
Galanthamine (**13**)	[39,40,41]	[32,41,42]		[41,43]	[32,35]	[41]	[36,41,42]	[30,41]	[13,28,32,37,38,41,42]	[41]		[41,44]	[34,41]	[45]
*O*-Demethyllycoramine-*N*-oxide (**14**)									[37]					
*O*-Demethyllycoramine (**15**)							[36]	[30,41]	[37,38,46]				[34]	
*N*-(Chloromethyl)lycoramine (**16**)		[31]						[30]						
Lycoramine-*N*-oxide (**17**)	[39]	[32]							[32,37,38]					
Lycoramine (**18**)	[39,41]	[32,41]		[41,43]	[32,35]	[41]	[36,41]	[41]	[32,37,38,41]	[41]		[41,44]	[34,41]	[45]
Norlycoramine (**19**)	[41]			[41]			[41]	[41]	[41]			[41]	[41]	
Narwedine (**20**)	[41]	[32]			[32,35]			[41]					[41]	
Sanguinine (**21**)		[41]		[41]			[36]	[30,41]	[37,46]				[34]	
***Galanthindole-type***														
Lycosinine B (**22**)												[44]		
***Haemanthamine-type***														
3α-Hydroxy-6β-acetylbulbispermine (**23**)									[47]					
3α-Methoxy-6β-acetylbulbispermine (**24**)									[47]					
3α,6β-Diacetylbulbispermine (**25**)									[47]					
6β-Acetyl-8-hydroxy-9-methoxycrinamine (**26**)									[48]					
6-Hydroxycrinamine (**27**)									[13]					
6β-Acetoxycrinamine (**28**)									[13,48]					
Haemanthamine (**29**)	[39]	[32,41]		[41]			[41]		[32,41]			[41,44]	[34]	
Haemanthidine (**30**)	[39]						[42]	[30]	[38]			[44]	[34,41]	
8-*O*-Demethylmaritidine (**31**)									[32,46]					
11-Hydroxyvittatine-*N*-oxide (**32**)		[32]												
11-Hydroxyvittatine (**33**)									[46]				[34]	
Vittatine (**34**)		[31,32]			[32]				[32,38]					
***Homolycorine-type***														
2α-Methoxy-6-*O*-ethyloduline (**35**)									[14,37]					
2α-Methoxy-6-*O*-methyloduline (**36**)		[49]			[32]				[14]					
2α-Hydroxy-6-*O*-methyloduline (**37**)	[39]	[31,32,49]							[13,32,37]					
2α-Hydroxy-6-*O-n*-butyloduline (**38**)		[31]												
2α-Hydroxyoduline (**39**)		[49]												
Oduline (**40**)		[32,49]			[32]				[14,32]					
2*α*-Hydroxy-8-*O*-demethylhomolycorine-*N*-oxide (**41**)									[48]					
8-*O*-Demethylhomolycorine-*N*-oxide (**42**)		[50]							[48]					
8-*O*-Demethylhomolycorine (**43**)						[41]			[14,38,41]	[41]				
9-*O*-Demethylhomolycorine (**44**)	[39]								[14,37]					
9-*O*-Demethyl-2*α*-hydroxyhomolycorine (**45**)									[37]					
8,9-Methylenedioxyhomolycorine-*N*-oxide (**46**)									[32,47]					
8-*O*-Acetylhomolycorine-*N*-oxide (**47**)									[13]					
Homolycorine-*N*-oxide (**48**)	[39]								[38,47]					
Homolycorine (**49**)	[39,40]	[31,49]				[41]			[13,14,38,41,42]	[41]		[44]		
*O-n*-Butyllycorenine (**50**)		[31]												
*O*-Ethyllycorenine (**51**)									[14,37]					
*O*-Methyllycorenine (**52**)		[31]							[14,37,38,41]			[44]		
*O*-Methyllycorenine-*N*-oxide (**53**)									[38]					
2α-Methoxy-6-*O*-methyllycorenine (**54**)												[44]		
Lycorenine (**55**)	[40]								[14,42]			[44]		
Radiatine (**56**)									[37]					
Hippeastrine (**57**)	[39]	[31,32,49]							[13,14,32,37,38,41]					
Hippeastrine-*N*-oxide (**58**)	[39]								[38]					
Unsevine (**59**)									[32]					
*Hostasinine-type*														
Hostasinine A (**60**)	[39]													
*Ismine-type*														
Ismine (**61**)													[34]	
*Lycorine-type*														
Assoanine (**62**)	[41]							[41]						
Caranine (**63**)	[41]	[41]		[41]				[41]	[41]	[41]		[41]	[41]	
Galanthine (**64**)	[41]	[41]		[41]		[41]	[36,41]	[41]	[46]			[41,44]	[41]	
Incartine (**65**)	[41]	[41]		[41]		[41]	[36,41]	[30]				[41]	[41]	
*N*-(Chloromethyl)ungiminorine (**66**)									[37]					
*N*-(Chloromethyl)narcissidine (**67**)								[30]				[44]		
Narcissidine (**68**)												[44]		
(−)-epi-Zephyranthine (**69**)									[37]					
Anhydrolycorine (**70**)	[41]	[41]		[41]		[41]	[41]	[41]		[41]		[41]	[41]	
Dihydrolycorine (**71**)		[32]			[32]				[13,37]					
11-Methoxylycorine (**72**)									[37]					
Pseudolycorine (**73**)					[35]				[37,51]				[34]	
5,6-Dehydrodihydrolycorine (**74**)	[41]								[13]					
5,6-Dehydrolycorine (**75**)									[32,47]					
11,12-Didehydroanhydrolycorine (**76**)		[41]		[41]		[41]	[41]	[41]	[41]	[41]		[41]	[41]	
2-Hydroxyanhydrolycorine-*N*-oxide (**77**)			[52]											
1-*O*-(3′-Hydroxybutanoyl)lycorine (**78**)														[45]
6-Oxodihydrolycorine (**79**)									[13]					
Lycorine (**80**)	[39,40,41]	[31,32,41,42]		[41,43]	[32,35]	[41]	[36,41,42]	[30,41]	[13,28,32,38,41,42]	[41]		[41,44]	[34,41]	[45]
1,2-Dihydroxy-anhydrolycorine-*N*-oxide (**81**)		[50]												
2-Hydroxy-6-oxoanhydrolycorine (**82**)		[50]												
1,2-Dihydroxy-6-oxoanhydrolycorine (**83**)		[50]												
Norpluviine (**84**)	[41]											[41]		
Pluviine (**85**)		[31,32]			[32]	[41]		[41]	[32,37]	[41]		[44]	[41]	
Hippadine (**86**)					[32]			[30]	[37]			[44]		
Lycosprenine (**87**)												[44]		
Sternbergine (**88**)														[45]
Tortuosine (**89**)												[44]		
1-Hydroxyungeremine (**90**)									[48]					
Ungiminorine (**91**)							[36]							[45]
Ungiminorine-*N*-oxide (**92**)							[36]							
*Montanine-type*														
Montanine (**93**)	[41]	[41]		[41]				[41]					[34,41]	
Pancratinine C/Squamigine (**94**)								[41]	[37]				[34]	
(−)-3-*O*-Menthylpancracine (**95**)									[37]					
Pancracine (**96**)									[37]					
Montabuphine (**97**)												[44]		
Lycolongirine C (**98**)								[30]						
*Narciclasine-type*														
7-Deoxynarciclasine/Lycoricidine (**99**)	[39]								[13]		[53]		[34]	[45]
Narciclasine/Lycoricidinol (**100**)	[39]								[16,37]		[53]		[34]	[45]
5,6-Dihydrobicolorine (**101**)									[13]			[44]		
Bicolorine (**102**)											[46]			
*N*-Methylcrinasiadine (**103**)								[30]						
*N*-Isopentylcrinasiadine (**104**)												[44]		
Crinasiadine (**105**)												[44]		
5,6-Dihydro-5-methyl-2-hydroxyphenanthridine (**106**)		[50]							[47]					
Trisphaeridine (**107**)								[30,41]	[46]			[41,44]		
*Tazettine-type*														
3-*O*-Ethyltazettinol (**108**)		[54]												
Deoxydihydrotazettine (**109**)									[28]					
Deoxypretazettine (**110**)								[30,41]	[28,41]					
Tazettine (**111**)	[41]	[41]		[41]		[41]		[30,41]	[38,41]	[41]		[41,44]	[34,41]	
6-*O*-Methylpretazettine (**112**)													[34]	
3-Epimacronine (**113**)									[28]					
3-Hydroxylatifaliumin C * (**114**)					[32]				[32]					
Dihydrolatifaliumin C * (**115**)		[32]			[32]				[32]					
Latifaliumin C * (**116**)		[32]												
*Other structural types*														
Norharmane (**117**)								[30]						
Harmane (**118**)								[30]						
Lycolongirine A (**119**)								[30]						
Perlolyrine (**120**)								[30]						
Colchicine (**121**)									[28]					
*N*-Methoxycarbonyl-2-demethylisocorydione (**122**)									[48]					
2-Demethylisocorydione (**123**)		[50]												
Isocorydione (**124**)		[50]												
8-Demethyldehydrocrebanine (**125**)		[50]												
*N*-Methoxycarbonyllindcarpine (**126**)			[52]											
*N*-Methoxycarbonylnandigerine (**127**)			[52]											
10-*O*-Methylhernovine-*N*-oxide (**128**)			[52]											

* Tentative identification based on mass spectra.

## 4. Studied Biological Activities of Extracts and Alkaloids Isolated from *Lycoris* Species

### 4.1. Antitumor Activity

In recent decades, AAs such as lycorine, pancratistatine, narciclassine, haemanthamine, and montanine have been intensively studied for their cytotoxic potential. All alkaloids displayed multiple properties towards various cancer cell lines including MOLT-4, HepG2, HeLa, MCF-7, CEM, K562, A549, Caco-2, HT-29, A2780 and others [55,56,57,58,59,60,61,62]. Thus, extracts and alkaloids isolated from different *Lycoris* species have been primarily tested for their cytotoxic activity on different cancerous cells. The dichloromethane extract (DCME) of bulbs of *L. aurea*, when tested both in vivo and in vitro against the murine sarcoma 180 cell line, using MTT assay [49], demonstrated promising inhibition effects on the cells in a dose-dependent manner. The in vivo study using sarcoma 180 bearing mice demonstrated inhibitory rates of 27.9% for a dosage of 20 µg/mL of DCME, 37.2% for a dosage of 40 µg/mL, and 53.5% for a dosage of 120 µg/mL [49].

Crude extract, DCME extract and pure alkaloids isolated from bulbs of *L. aurea* were also evaluated for their antiproliferative activities against the SH-SY5Y cell line. The crude and DCME extracts revealed cytotoxicity at concentrations of 5 µg/mL [31]; of the pure alkaloids, only lycorine (**80**) demonstrated significant cytotoxicity at a concentration 6.25 µM [31].

Alkaloidal extracts of three *Lycoris* species were screened for their cytotoxic potential against HepG2 cells at a concentration of 10 µg/mL with inhibitory rates of 78.0%, 84.9%, and 66.8% for *L. aurea*, *L. radiata* and *L. guangxiensis*, respectively [32].

An alcoholic extract of fresh bulbs of *L. albiflora* showed promising cytotoxic activity against HL-60 cells, with an IC_50_ value of 1.7 µg/mL [39]. This resulted in a detailed phytochemical study being undertaken to isolate pure AAs and test them for their cytotoxic activity; fifteen AAs were isolated (Table 1), which were tested for their cytotoxic activity against the cancerous cell line HL-60. The most potent AAs were also subjected to a cytotoxic screening against HSC-2 cells [39]. Narciclassine-type alkaloids 7-deoxynarciclasine (**99**; also known as lycoricidine) and narciclasine (**100**, also known as lycoricidinol) induced apoptosis in both HL-60 and HSC-2 cells. Moreover, narciclasine (**100**) induced transient autophagy and morphological changes in mitochondria in the early stages of the apoptotic cell death process in HSC-2 cells [39]. Within previous studies, narciclasine (**100**) exhibited potent in vitro cytotoxicity against various cancer cells and showed great potential against primary brain cancers, as well as brain metastases in vivo [63,64,65]. Within the latest study, narciclasine (**100**) displayed preferential cytotoxicity towards primary effusion lymphoma cell lines (PEL), an aggressive type of non-Hodgkin lymphoma, with IC_50_ values ranging from 7 to 14 nM [66]. 7-Deoxynarciclasine (**99**) displayed approximately 10 times lower cytotoxicity against the tested PEL cell lines (IC_50_ = 82–162 nM) [66]. Previous in vitro studies frequently focused on the cytotoxicity of narciclasine against fibroblast (IC_50_ = 7.5 µM) and cancer cells (IC_50_ = 30 nM), which indicated the compound‘s selectivity to cancer cells and only higher concentrations affected the viability of fibroblasts [67]. On the other hand, it was reported that narciclasine (**100**) showed only modest anti-tumor effects in mice in vivo, with considerable toxicity [68]. Thus, narciclasine (**100**) has not been tested in human clinical trials up to now. The inhibitory effects on L02 (human normal liver cell line) and murine macrophages RAW264.7 indicated that narciclasine (**100**) might have significant side effects, and, therefore, further studies are urgently needed [16]. Narciclasine (**100**) has also been shown to inhibit the cytotoxicity of calprotectin in rat adjuvant arthritis mode, and several studies have reported that narciclasine (**100**) exhibits strong anti-inflammation activity in vitro and in vivo [69]. LPS-stimulated RAW264.7 cells were employed to investigate the anti-inflammatory effects of narciclasine (**100**) in order to explore its underlying mechanism [16]. Recently, narciclasine was named ‘Molecule of the Week’ by the American Chemical Society (ACS) for its potential as a cancer drug [66].

The in vitro antiproliferation assay of hippeastrine (**57**) isolated from fresh bulbs of *L. radiata* revealed strong inhibition of proliferation of HT-29 and Hep G2 cells in an intuitive dose-dependent manner, with IC_50_ values of 3.98 ± 0.29 µg/mL and 11.85 ± 0.20 µg/mL, respectively [12]. The results of the cytotoxic studies of AAs isolated from *Lycoris* species are summarized in the following table (Table 2).

### 4.2. Biological Activity Connected with Alzheimer’s Disease

AD is a major neurodegenerative illness, and is the major cause of dementia associated with aging [70]. The current treatment of AD is only symptomatic and mainly involves restoring of acetylcholine (ACh) levels through acetylcholinesterase (AChE) inhibition [71]. Three AChE inhibitors, namely donepezil, galanthamine (**13**) and rivastigmine, are currently used as the main therapeutic option for AD treatment [72]. Since the Amaryllidaceae alkaloid galanthamine (**13**) has been introduced into clinical practice, other Amaryllidaceae alkaloids have received attention as potential AChE inhibitors [5,73,74,75,76]. Surprisingly, only a few AAs isolated from *Lycoris* plants have been studied in terms of their AChE inhibition potential. AAs isolated from bulbs of *L. longituba* (Table 1) have been screened for their AChE inhibition effects. The most active alkaloids in the AChE assay belong to the galanthamine structural type of AAs, which was in agreement with previous results. The best AChE inhibition activity has been obtained for galanthamine (IC_50_ = 2.43 ± 0.66 µM), which is already used in the therapy of AD as a competitive, reversible, selective inhibitor of AChE. Promising AChE inhibition activities were obtained also for further AAs of galanthamine-type (Table 3), and, interestingly, for *N*-methylcrinasiadine (**103**; IC_50_ = 4.23 ± 1.13 µM) and deoxypretazettine (**110**; IC_50_ = 8.44 ± 0.83 µM). Moreover, isolated AAs were tested for their neuroprotective effects against CoCl_2_, H_2_O_2_ and Aβ_25–35_-induced neuronal cell death in dopaminergic neuroblastoma SH-SY5Y cells [30]. Incartine (**65**), trisphaeridine (**107**), *N*-(chloromethyl)galanthamine (**12**), sanquinine (**21**), *O*-demethyllycoramine (**15**), and deoxypretazettine (**110**) showed significant neuroprotective effects against all three injury models [30]. Lycolongirine C (**103**) and *N*-methylcrinasiadine (**110**) exhibited significant neuroprotective activities against H_2_O_2_ and Aβ_25–35_-induced cell death [30].

The crude and DCME extracts obtained from the bulbs of *L. aurea* were also tested for their neuroprotective effects against CoCl_2_, and H_2_O_2_-induced cell injuries in SH-SY5Y cells [31]. Both extracts exhibited modest neuroprotective effects against CoCl_2_-induced SH-SY5Ycell injury, but a significant effect in H_2_O_2_-induced SH-SY5Y cell injury [31]. The mentioned extracts were further subjected to column chromatography and separation processes, resulting in the isolation of thirteen AAs (Table 1), which were also tested for their neuroprotective effects. Alkaloids belonging to the homolycorine- and galanthamine-type (**9**, **12**, **13**, **16**, **37**, **38**, **50**, and **52**) exhibited significant neuroprotective effects against CoCl_2_-induced SH-SY5Y cell injury, while alkaloids **9**, **12**, **16**, **37**, **38**, **50**, **53**, and **85** showed obvious neuroprotective effects against H_2_O_2_-induced SH-SY5Y cell death [31].

Seven AAs isolated from *L. sprengeri* were evaluated for their neuroprotective activities against the same models [44]. *O*-methyllycorenine (**52**), and hippadine (**86**) exhibited significant neuroprotective effects against H_2_O_2_-induced SH-SY5Y cell death; lycosprenine (**87**), *O*-methyllycorenine (**52**), and tortuosine (**89**) showed obvious neuroprotective effects against CoCl_2_-induced SH-SY5Y cell injury [44]. The results obtained within the mentioned studies indicate that compounds of the same structural type of AAs (e.g., homolycorine, lycorine and galanthamine) may have potential for further development as neuroprotective compounds.

### 4.3. Antimalarial Activity

Malaria is one of the most common vector-borne infectious diseases. This disease is caused by protozoan parasites of the genus *Plasmodium* [77]. Alkaloids isolated from *L. radiata* were evaluated in vitro for their antimalarial activity using the drug-resistant D-6 strain and drug sensitive W-2 strain of *P. falciparum* [47]. Within the tested AAs, only 5,6-dehydrolycorine (**75**) exhibited antimalarial activity, with IC_50_ values of 2.3 µM for the D-6 strain and 1.9 µM for the W-2 strain of *P. falciparum* [47]. Other studied AAs displayed only weak or no antimalarial activity against the studied strains (Table 4).

### 4.4. Further Studied Biological Activities

Alkaloids 2*α*-methoxy-6-*O*-ethyloduline (**35**), 2*α*-methoxy-6-*O*-methyloduline (**36**) and hippeastrine (**57**), all isolated from the bulbs of *Lycoris radiata*, showed weak antiviral activities against flu virus A with IC_50_ values of 2.06, 0.69 and 2.71 µg.mL^-1^ and CC_50_ values of 14.37, 4.79, and 80.12 µg.mL^−1^ [14], respectively.

Aphids are one of the most destructive and economically important pests of plants on earth and extensive use of insecticides has resulted in the development of insecticide resistance among aphids across regions [78]. Thus, the insecticidal activity of ten AAs isolated from *L. radiata* against *Aphis citricola* has been studied [28]. LD_50_ values were measured by a capillary drip method and nine of the tested AASs displayed aphicial activity. *N*-Allylnorgalanthamine (**10**) possessed the highest aphicial activity (LD_50_ = 4.92 ± 0.83 ng/aphid), which was comparable with the commercial pesticide methomyl (LD_50_ = 2.91 ± 0.18 ng/aphid). Inhibition of AChE is a main target enzyme for many insecticides (especially carbamates and organophosphates) [79]; the in vitro inhibition of AChE of *N*-allylnorgalanthamine (**10**) has also been studied. This compound demonstrated remarkable inhibition activity against AChE in *A. citricola* with a value of IC_50_ = 2.1 nM.

## 5. Conclusions

In conclusion, this review summarizes the ethnobotanical, phytochemical, and pharmacological information about plants and AAs of the genus *Lycoris*. So far, fourteen *Lycoris* species have been phytochemically studied, and nearly 120 AAs of different structural types have been either identified or isolated. The presence and structures of some reported AAs must be reevaluated, as they may be isolation artifacts, and not naturally occurring compounds. *Lycoris* plants are above all a rich source of homolycorine- and lycorine-type AAs. Most of the isolated AAs have been studied for different biological activities with impact on antitumor and neuroprotective activities. The antimalarial, antiviral and insecticidal activities of some AAs have also been described. In the light of the presented overview of scientific data, the genus *Lycoris* can be recognized as an interesting source of different structural types of AAs with a wide range of biological activities.

## Figures and Tables

**Table 2:** Impact of Amaryllidaceae alkaloids (AA) isolated from *Lycoris* plants on proliferation of cancer and non-cancer cells using in vitro assays. Results are expressed as IC_50_ values in µM, unless otherwise stated.

Alkaloid (No.)	Cell Line										Ref.
	HL-60	A549	MCF-7	BEN-MEN-1	CCF-STTG1	CHG-5	SHG-44	U251	SMMC-7721	W480	
3α-Hydroxy-6β-acetylbulbispermine (**23**)	7.1 ± 0.9			>100	29.4 ± 4.1	29.4 ± 5.3	28.3 ± 2.7	15.8 ± 1.7	66.8 ± 9.4	53.5 ± 12.4	[47]
3α-Methoxy-6β-acetylbulbispermine (**24**)	8.6 ± 1.4			>100	29.7 ± 5.4	29.6 ± 6.3	29.1 ± 3.8	16.7 ± 2.6	68.2 ± 12.3	50.1 ± 7.8	[47]
3α,6β-Diacetylbulbispermine (**25**)	7.3 ± 1.1			>100	27.1 ± 5.1	30.1 ± 4.4	27.1 ± 3.2	17.4 ± 2.1	63.2 ± 11.8	51.1 ± 10.9	[47]
6β-Acetyl-8-hydroxy-9-methoxycrinamine (**26**)	8.6 ± 1.4			>100	29.4 ± 4.1	29.6 ± 5.3	27.1 ± 3.2	17.4 ± 2.1	68.2 ± 12.3	53.5 ± 12.4	[48]
6β-Acetoxycrinamine (**28**)	8.1	24.3	15.0								[13]
8,9-Methylenedioxyhomolycorine-*N*-oxide (**46**)	>100			>100	83.2 ± 13.7	>100	>100	>100	86.2 ± 17.4	>100	[47]
8-*O*-Acetylhomolycorine-*N*-oxide (**47**)	>40	>40	>40								
Homolycorine-*N*-oxide (**48)**	>100			>100	>100	93.0 ± 21.1	>100	>100	85.0 ± 16.2	>100	[47]
5,6-Dehydrodihydrolycori-ne (**79**)	>40	>40	>40								[13]
5,6-Dehydrolycorine (**75**)	10.8 ± 1.6			>100	10.3 ± 0.9	10.2 ± 1.6	9.4 ± 1.3	11.8 ± 0.7	10.5 ± 0.9	11.6 ± 1.1	[47]
2-Hydroxy-anhydrolycorine-*N*-oxide (**77**)				>100	>100	>100	>100	93.7			[52]
1-Hydroxyungeremine (**90**)	10.8 ± 1.6			>100	10.3 ± 0.9	10.2 ± 1.6	9.4 ± 1.3	11.8 ± 0.9	10.5 ± 0.9	11.6 ± 1.1	[48]
7-Deoxynarciclasine/Lyco-ricidine (**99**)	0.15										[39]
Narciclasine/Lycoricidi-nol (**100**)	0.018										[39]
5,6,-Dihydro-5-methyl-2-hydroxyphenanthridine (**106**)	81.3 ± 15.7			>100	>100	>100	>100	>100	>100	>100	[47]
**Alkaloid (No.)**	**HT-29**	**HepG-2**	**SK-OV-3**	**SCL-1**	**CAL-27**	**UMSCC-1**	**Detroit-562**	**SCC-PKU**	**TCA-83**	**HSC-2**	**Ref.**
8-*O*-Demethylhomolycorine-*N*-oxide (**42**)		11.6		13.2	12.3	12.3	12.9	13.2	16.7		[50]
Hippeastrine (**57**)	12.6 ± 0.92	37.62 ± 0.63									[12]
2-Hydroxy-anhydrolycorine-*N*-oxide (**77**)		67.7	76.2								[52]
**Alkaloid (No.)**	**HT-29**	**HepG-2**	**SK-OV-3**	**SCL-1**	**CAL-27**	**UMSCC-1**	**Detroit-562**	**SCC-PKU**	**TCA-83**	**HSC-2**	**Ref.**
1,2-Dihydroxy-anhydrolycorine-*N*-oxide (**81**)		>100		>100	>100	>100	94.3	>100	>100	>100	[50]
2-Hydroxy-6-oxoanhydrolycorine (**82**)		>100		>100	>100	>100	>100	95.5	91.2		[50]
1,2-Dihydroxy-6-oxoanhydrolycorine (**83**)		>100		>100	>100	>100	>100	88.3	91.8		[50]
7-Deoxynarciclasine/Lyco-ricidine (**99**)										1.7 ± 0.2	[39]
Narciclasine/Lycoricidi-nol (**100**)	1.373	0.08								0.05	[16,39]
5,6,-Dihydro-5-methyl-2-hydroxyphenanthridine (**106**)		>100		>100	>100	>100	>100	87.6	>100		[50]

HL-60 (acute promyelocytic leukemia); A549 (lung carcinoma); MCF-7 (breast carcinoma); BEN-MEN-1 (meningioma); CCF-STTG1 (astrocytoma); CHG-5 (glioma); SHG-44 (glioma); U251 (glioma); SMMC-7721 (hepatocellular carcinoma); W480 (colon cancer); HT-29 (colon carcinoma); Hep G2 (liver cancer); SK-OV-3 (ovarian carcinoma); SCL-1 (squamous carcinoma); CAL-27 (squamous carcinoma); UMSCC-1 (squamous carcinoma); Detroit-562 (pharyngeal carcinoma); SCC-PKU (squamous carcinoma); TCA-83 (squamous carcinoma); HSC-2 (human squamous carcinoma).

**Table 3:** AChE inhibitory activity of Amaryllidaceae alkaloids isolated from bulbs of *L. longituba.* [30].

Alkaloid (No.)	% AChE Inhibition (100 µM)	IC_50_ (µM)
11β-Hydroxygalanthamine (**8**)	96 ± 0	3.04 ± 0.61
*N*-Norgalanthamine (**9**)	92 ± 1	2.76 ± 0.65
*N*-(Chloromethyl)galanthamine (**12**)	94 ± 1	5.55 ± 0.63
Galanthamine (**13)**	95 ± 1	2.43 ± 0.66
*O*-Demethyllycoramine (**15**)	86 ± 1	8.13 ± 1.49
*N*-(Chloromethyl)lycoramine (**16**)	79 ± 1	25.76 ± 1.09
Sanguinine (**21**)	93 ± 0	5.30 ± 0.76
Haemanthidine (**30**)	39 ± 1	208.10 ± 1.58
Incartine (**65**)	41 ± 1	148.70 ± 1.46
*N*-(Chloromethyl)narcissidine (**67**)	36 ± 3	190.70 ± 2.00
Lycorine (**80**)	32 ± 2	224.80 ± 3.01
Hippadine (**86**)	42 ± 2	117.60 ± 1.79
Lycolongirine C (**98**)	45 ± 2	194.80 ± 2.31
*N*-Methylcrinasiadine (**103**)	85 ± 1	4.23 ± 1.13
Trisphaeridine (**107**)	33 ± 1	190.70 ± 2.00
Deoxypretazettine (**110**)	89 ± 0.38	8.44 ± 0.83

**Table 4:** In vitro antimalarial activity of Amaryllidaceae alkaloids isolated from *L. radiata* against two *Plasmodium falciparum* strains [47].

Alkaloid (No.)	D-6 IC_50_ (µM)	W-2 IC_50_ (µM)
3α-Hydroxy-6β-acetylbulbispermine (**23**)	17.9	19.3
3α-Methoxy-6β-acetylbulbispermine (**24**)	21.3	23.4
3α,6β-Diacetylbulbispermine (**25**)	18.9	20.1
8,9-Methylenedioxyhomolycorine-*N*-oxide (**46**)	>100	>100
Homolycorine-*N*-oxide (**48**)	>100	>100
5,6-Dehydrolycorine (**75**)	2.3	1.9
5,6,-Dihydro-5-methyl-2-hydroxyphenanthridine (**106**)	>100	>100
Chloroquine *	9.8 ^a^	6.7 ^a^

^a^ Chloroquine data are expressed as IC_50_ values in nM; * standard.
